# Arcuate fasciculus cortico-cortical evoked potentials and direct cortico-subcortical stimulation during awake craniotomy for debulking of left dominant temporal oligodendroglioma

**DOI:** 10.3171/2024.10.FOCVID24126

**Published:** 2025-01-01

**Authors:** Ryan P. Hamer, Santosh Poonnoose

**Affiliations:** 1 Department of Neurosurgery, St. Vincent’s Hospital Melbourne, Victoria;; 2 Faculty of Medicine, Health & Dentistry, University of Melbourne, Victoria;; 3 Faculty of Medicine & Health, University of Sydney, New South Wales;; 4 Flinders Health and Medical Research Institute, College of Medicine and Public Health, Flinders University, Adelaide, South Australia; and; 5 Department of Neurosurgery, Flinders Medical Centre, Bedford Park, South Australia, Australia

**Keywords:** brain mapping, neuro-oncology surgery, glioma surgery, arcuate CCEPs, language mapping, direct cortical stimulation

## Abstract

Electrophysiological mapping and monitoring techniques permit the objective measurement of eloquent cortical regions and accompanying white matter tracts to reduce the incidence of iatrogenic injury in glioma surgery. Recently, there has been increased interest in mapping and monitoring of the human arcuate fasciculus via cortico-cortical evoked potentials (CCEPs) during awake and asleep craniotomy. The authors present the case of a 27-year-old female who underwent a hypnosis-assisted awake craniotomy with cortico-subcortical language mapping and arcuate fasciculus CCEPs. There were no adverse effects associated with CCEP monitoring. Further investigation is required in order to substantiate the technique and validate clinical significance criteria, which may reliably permit asleep language mapping and monitoring.

The video can be found here: https://stream.cadmore.media/r10.3171/2024.10.FOCVID24126

## Transcript

Clinical approaches and philosophies are widely heterogeneous with regard to intra-axial supratentorial glioma surgery; however, it is universally accepted that safe and maximal resection with the avoidance of significant ischemic or neurological injury is considered the primary goal.

Advances in electrophysiological mapping and monitoring techniques permit the objective measurement of eloquent motor and language cortical regions and accompanying white matter tracts to reduce the incidence of iatrogenic neurological injury in glioma surgery.^1^

Various electrophysiological biomarkers exist that can be reliably mapped and monitored in both awake and asleep craniotomy, which are often integrated in a multimodal fashion.^2^

### 1:04 Contemporary Brain Mapping.

Subject to the clinical indication, this usually consists of bipolar low-frequency direct cortico-subcortical stimulation (bDCS) for cognitive mapping, monopolar high-frequency DCS for motor mapping (mDCS), direct cortical MEPs via a subdural electrode for continuous corticospinal tract monitoring (sMEP), sensorimotor phase reversal for delineation of pericentral anatomy (SPR), and electrocorticography or intracranial electroencephalography for the detection of afterdischarges associated with cortical stimulation or mapping of epileptogenic irritative zones (ECoG).

Recently, there has been increased interest in mapping and monitoring of the human arcuate fasciculus in awake and asleep settings, which represents an emerging frontier in neuro-oncology surgery, including the prospect of monitoring expressive language function under general anesthesia.^3,4^

### 2:00 Clinical Case.

Here we present our initial experience in exploring feasibility and clinical utility of arcuate CCEPs in a 27-year-old female who presented with generalized seizure activity, auditory hallucinations, and baseline semantic paraphasias.

Preoperative MRI revealed a suspected diffuse lower-grade glioma comprising the anterior and middle temporal lobe.

The patient was scheduled for a hypnosis-assisted awake craniotomy with bipolar low-frequency cortico-subcortical stimulation with electrocorticography (ECoG) and arcuate fasciculus cortico-cortical evoked potentials (CCEPs).

The patient’s hypnotic state was established in the anesthetic bay by a senior consultant anesthetist with an extensive history in clinical hypnosis. This involved a series of mindfulness techniques as part of a prospective study we have initiated to explore electrophysiological biomarkers associated with cortical excitability changes and the subsequent impact on awake craniotomy compliance.

The procedure was performed with the patient in the supine position with a lateral head tilt with the head fixated.

Targets were applied via stereotactic navigation in addition to posterior margin, temporal stem, and temporal horn fence posting.

### 3:19 Direct Cortical Stimulation Language Mapping.

Following craniotomy and dural opening, a 4-channel subdural electrode (inomed Medizintechnik GmBH) was positioned toward the inferior frontal gyrus/pars triangularis. Another 4-channel subdural electrode was introduced to the posterior superior temporal gyrus (WISE Srl). Both electrodes were originally configured for ECoG recording during language mapping to mitigate the risk of intraoperative seizures (inomed ISIS, inomed Medizintechnik GmBH).

Extensive cortical mapping via bDCS then proceeded at 2 mA, and increased in 1-mA intervals until there was evidence of speech and language disturbance as indicated by the speech pathology team, or if there were afterdischarge activity as recorded via ECoG. No such activity was recorded. Bipolar DCS toward the inferior frontal gyrus electrode was positive for speech arrest at 3 mA, which would be used for CCEP stimulation.

### 4:20 CCEP Findings.

Optimization of arcuate CCEPs was performed in conjunction with Seidel et al.’s recent study.^4^ The surgical team recognized that CCEP morphology would not influence surgical decision-making due to lack of clinical evidence relating to signal change.

CCEP stimulation was initially conducted very conservatively at low intensities (3 mA) and ramped up progressively until 20 mA, where a positive N1/P1/N2 morphology was observed. Stimulation was conducted with 2 adjacent contacts of the stimulating strip in a bipolar stimulation. CCEP1 refers to bipolar stimulation at channels 1 and 2, and CCEP3 refers to bipolar stimulation at channels 3 and 4. The CCEP recording electrode was configured in both bipolar and referential montages for analysis. For referential recordings, the contralateral mastoid was used as a reference.

Stimulation was carried out with alternating monophasic square waves of constant-current, single-pulse electrical stimulation, with a pulse duration of 0.5 msec, a stimulation intensity of 5–20 mA, and a stimulation frequency of 1.1 Hz. The recordings were acquired with a hardware high-pass filter of 30 Hz, a hardware low-pass filter of 5 kHz, an epoch of 1000 msec, and a sampling rate of 20,000 Hz. Software filters were set at 10 for high-pass and 1500 for low-pass, while the sensitivity was 50–300 μV/Div and the sweep was 200–300 msec. Trials of 30 averages each were established.

As per Seidel et al.’s recent study,^4^ N1 was defined as a negative deflection occurring at a peak latency between approximately 10 and 30 msec and with a duration of at least 10 msec, as determined by visual inspection. A CCEP was defined as a signal containing a clear and reproducible N1.

### 6:37 Tumor Resection and Subcortical Language Mapping.

Resection continued posteriorly and posterosuperiorly, when subcortical mapping via bDCS at 5 mA contributed to object naming issues. Continued resection to floor of middle fossa and temporal pole, resecting the entirety of the middle and inferior temporal gyri. A small portion of posterior superior temporal gyrus remained preserved. Resection then followed the lateral wall of temporal horn, debulking inferiorly. Medial temporal lobe structures were not resected. Histopathological findings returned oligodendroglioma WHO grade 2.

### 7:07 Conclusions.

There were no adverse effects associated with CCEP stimulation or recording, nor did the intermittent nature of testing bear any language interruption or untoward effects for the patient during regular speech assessments. This is due to the reduced pulse width in comparison to classical bipolar low-frequency DCS, which contributes toward transient cognitive inhibition.

Further investigation is required with regard to clinical significance criteria to examine whether the technique will join the electrophysiological armamentarium routinely endorsed in contemporary neuro-oncology surgery. However, network-level electrophysiological biomarkers such as the arcuate fasciculus CCEP may provide opportunities for asleep language mapping and monitoring, in addition to other white matter networks associated with cognitive function, which is particularly encouraging for patients not indicated for awake craniotomy.

Hypnosis-assisted craniotomy may also permit more extensive intraoperative language assessment possibly in conjunction with electrophysiological assessment and optimization of the hypnotic state via ECoG and evoked potentials.^5^

## Disclosures

The authors report no conflict of interest concerning the materials or methods used in this study or the findings specified in this publication.

## Author Contributions

Primary surgeon: Poonnoose. Editing and drafting the video and abstract: Hamer. Critically revising the work: Hamer. Reviewed submitted version of the work: Hamer. Approved the final version of the work on behalf of both authors: Hamer. Neurophysiologist: Hamer.

## Supplemental Information

### Patient Informed Consent

The necessary patient informed consent was obtained in this study.
